# Draft genome sequence of *Enterococcus faecium* SP15, a potential probiotic strain isolated from spring water

**DOI:** 10.1186/s13104-019-4136-0

**Published:** 2019-02-22

**Authors:** Fauzia Aziz, Muhammad Nasim Khan, Safia Ahmed, Simon Colin Andrews

**Affiliations:** 1 Department of Zoology, The University of Azad Jammu and Kashmir, Muzaffarabad, Azad Kashmir Pakistan; 2 School of Biological Sciences, University of Reading, Reading, RG6 6AJ UK; 3 Department of Zoology, The University of Poonch, Rawalakot, Azad Kashmir Pakistan; 4 Department of Microbiology, Quaid-I-Azam University, Islamabad, 45230 Pakistan

**Keywords:** *Enterococcus faecium*, Probiotic, Draft genome assembly, Enterocin, Spring water

## Abstract

**Objectives:**

Enterococci are Gram-positive lactic acid bacteria and common inhabitants of the gastrointestinal tract of mammals, including humans. They are also widely distributed in diverse environments such as soil, water, vegetables and food. *Enterococcus faecium* is able to produce antimicrobial compounds (enterocins) and thus can act as a probiotic. *E. faecium* SP15 is a newly identified enterocin-producing strain from spring water that has been subjected to genome sequence analysis to provide understanding of its antimicrobial and probiotic properties.

**Data description:**

The draft genome of *E. faecium* SP15 comprises of 2,783,033 bp with a G+C content of 38.08%. Five genetic loci predicted to specify enterocin production were identified, but no virulence factors could be detected and only two potential antibiotic resistance genes were noted.

## Objective

Enterococci are Gram-positive lactic acid bacteria with a wide environmental distribution, encompassing many species from a wide variety of ecological niches [1]. *Enterococcus faecium* is a major nosocomial pathogen often causing neonatal meningitis or endocarditis [2]. However, certain strains of *E. faecium* have beneficial effects on human health due to their probiotic activity [3]. For example, *E. faecium* T-110 is a syndicate member in several probiotic products including BIO-THREE^R^ which is widely prescribed for human, animal and aquacultural use [3] and *E. faecium* strain L-3 is the principle organism in the probiotic Laminolact [4]. *E. faecium* is well known for its ability to produce bacteriocins, but there are relatively few reports in the literature on the genome sequence of *E. faecium* from non-clinical sources [5, 6] and there are no current NCBI database genome entries for *E. faecium* isolated from natural water sources.

## Data description

*Enterococcus faecium* SP15 was isolated from spring water in Rawalakot (Azad Kashmir, Pakistan), a site of relative isolation with little waste water contamination. The strain exhibited strong antimicrobial activity against a panel of seven indicator strains, including *Listeria monocytogenesis*, indicative of enterocin production. *E. faecium* SP15 genomic DNA was extracted using a GeneJET genomic DNA purification kit (Thermofisher Scientific) as recommended by the vendor and assessed using a NanoDrop ND-1000 spectrophotometer and 0.7% agarose gel electrophoresis. Genome sequencing was performed by MicrobesNG (University of Birmingham, UK) using Illumina MiSeq and HiSeq 2500 platforms (Illumina, UK) with 2 × 250 bp paired-end reads. The reads were trimmed using Trimmomatic version 0.30 [7] and the quality was assessed using in-house scripts combined with BWA-MEM software 0.7.16 [8]. De novo assembly was performed with SPAdes software version 3.9.0 [9] and assembly metrics were calculated using QUAST version 2.0 [10]. Gene prediction and annotation were carried out using the Pathosystems Resources Integration Center (PATRIC) web server [11], RAST version 2.0 [12] and the NCBI, PGAP version 4.6 [13]. The tRNA genes were predicted by tRNA scan-SE 2.0 [14]. Antimicrobial mechanisms (e.g. enterocin production) were explored with BAGEL 3 [15] and anti-SMASH V4 [16]. Virulence factors were identified using the virulence factor database, VFDB [17]. Antibiotic resistance gene were identified using the Comprehensive Antibiotic Resistance Database, CARD [18], and acquired resistance genes were predicted by Resistance Finder 3.0 [19]. Contigs were ordered by alignment against the most closely related sequence in GenBank [20] (*E. faecium* T110, CP006030; 99% identity) using progressive Mauve version 2.4.0 [21]. Intact and incomplete prophage regions were identified through the integrated search and annotation tool, PHAST [22]. Clustered regularly interspaced short palindromic repeat (CRISPR) arrays were identified using CRISPR finder [23].

The draft genome assembly consisted of 121 contigs with a total size of 2,783,033 bp (Table 1). The genome sequence data was at 30× coverage with an N50 of 102,590 bp and mean GC content 38.08%. A total of 2900 protein-encoding genes were predicted of which 2063 were assigned putative functions while 837 remain hypothetical. A total of 63 tRNA structural genes were identified. BAGLE 3 predicted five bacteriocin biosynthetic gene clusters (enterocin-HF, enterocin-P, enterocin SE-K4, enterocin L50A/L50B and enterolysin). No virulence factors (AS, Ace, Acm, Scm, EfaA, EcbA, Esp, Cyl, GelE and SprE) were detected. Two antibiotic resistance genes *aac* (6′)-li and *msrC* (98 and 97% identity, respectively) were identified conferring resistance to aminoglycosides, and macrolides and streptogramin B antibiotics, but no acquired resistance determinants were found. Four prophage loci were predicted of which three were intact (Strept_9871, Lactob_phig1e and Staphy_SPbeta (40, 36.9 and 32.7 kb, respectively) and one incomplete (Salmon_SJ46, 17.4 kb). One CRISPR array was identified of 190 bp, containing three spacers with a highly conserved 24 bp DR region, and although two *cas* gene clusters (*cas*3_typeI, *cas*4_typeI-II) were found, these were not associated with the CRISPR array.

**Table 1:** Overview of data files

Label	Name of data file/data set	File types (file extension)	Data repository and identifier (DOI or accession number)
Data file	Whole genome shotgun project	FASTA	DDBJ/ENA/GenBank (accession RDQA00000000)

Two major replicons are apparent: a chromosome of ~ 2,545,000 bp and a plasmid of ~ 149,300 bp (related to plasmid pNB2354 from *E. faecium* NRRL-B-2354, CP004064).

## Limitations

Current data is based on the draft level genome such that the exact length of the genome, and the number of rRNA genes and repetitive elements, cannot be absolutely determined. Furthermore, the genome includes extrachromosomal elements that cannot be predicted precisely.

## Abbreviations

PGAP: prokaryotic genome annotation pipeline
BAGEL 3: BActeriocin GEnome mining tooL version 3
antiSMASH: antibiotic and secondary Metabolite Analysis SHell
